# Supplementary material for: Human cytomegalovirus infection induces L1 expression through UL38-dependent mTOR-KAP1 pathway
Source: PLoS One. 2025 Apr 23;20(4):e0320512. doi: 10.1371/journal.pone.0320512 (PMC12017509; doi:10.1371/journal.pone.0320512)

Figure 1A

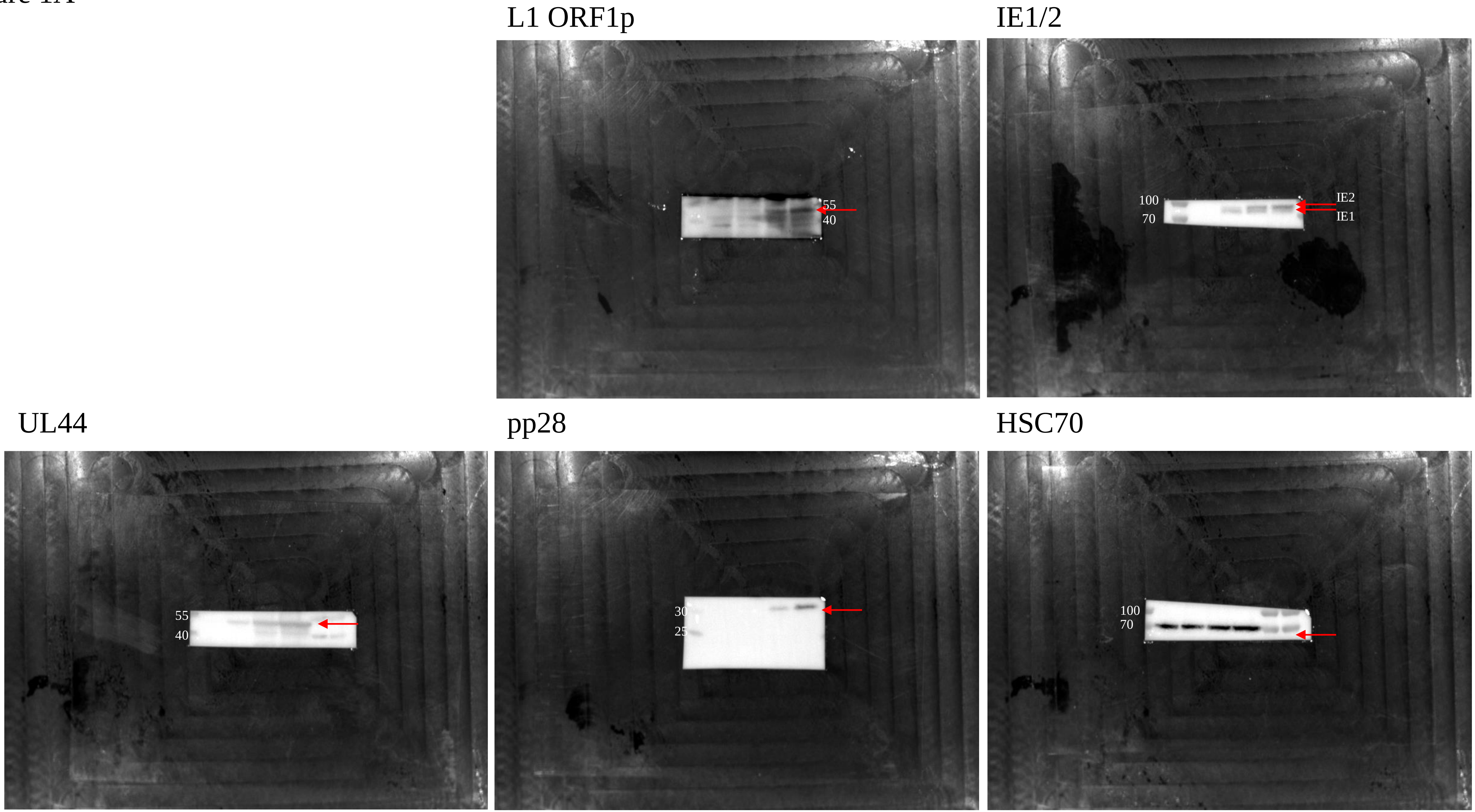
L1 ORF1p
55
40
IE1/2
100
70
IE2
IE1
UL44
55
40
pp28
30
25
HSC70
100
70

Figure 1B

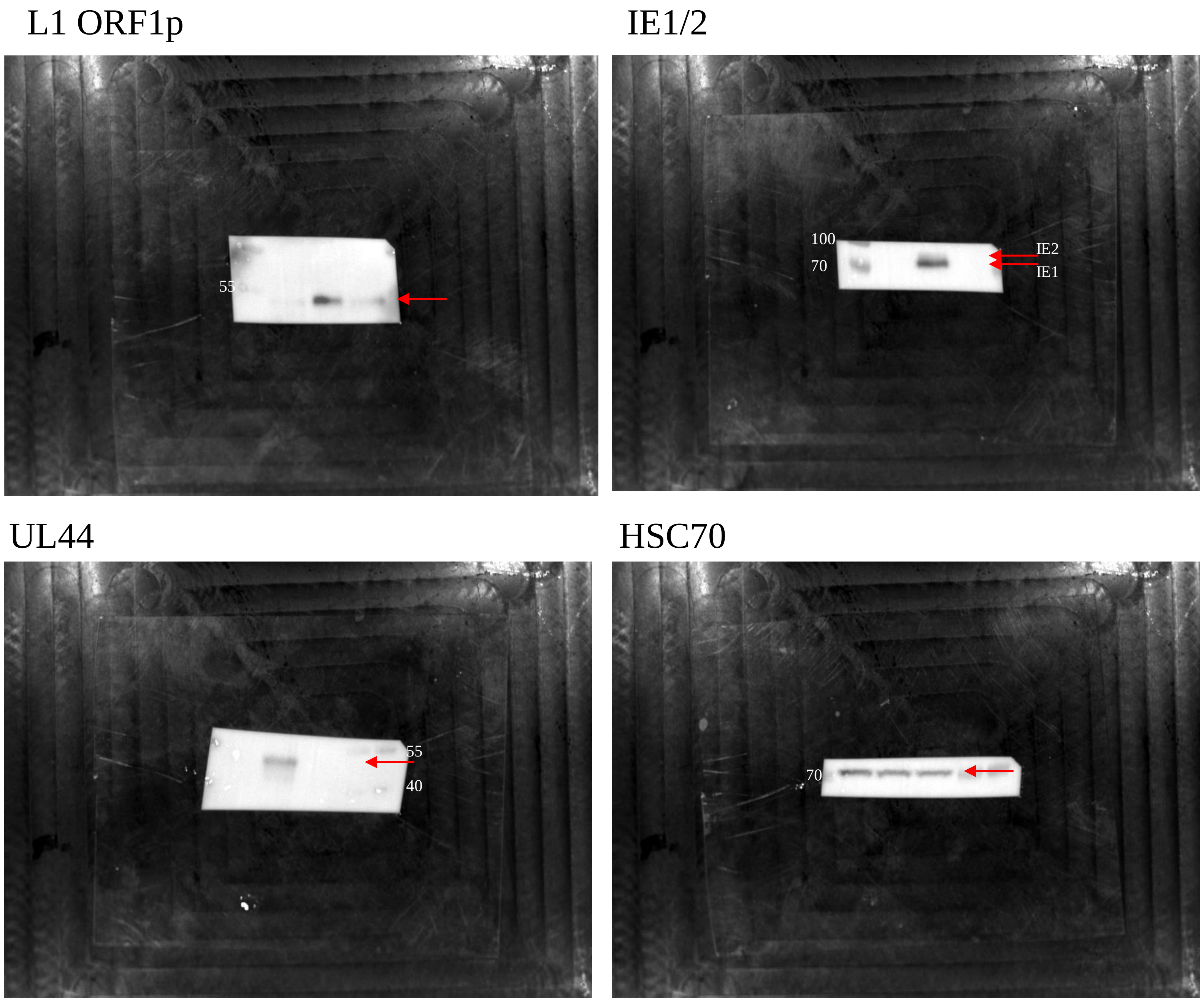

Figure 2A

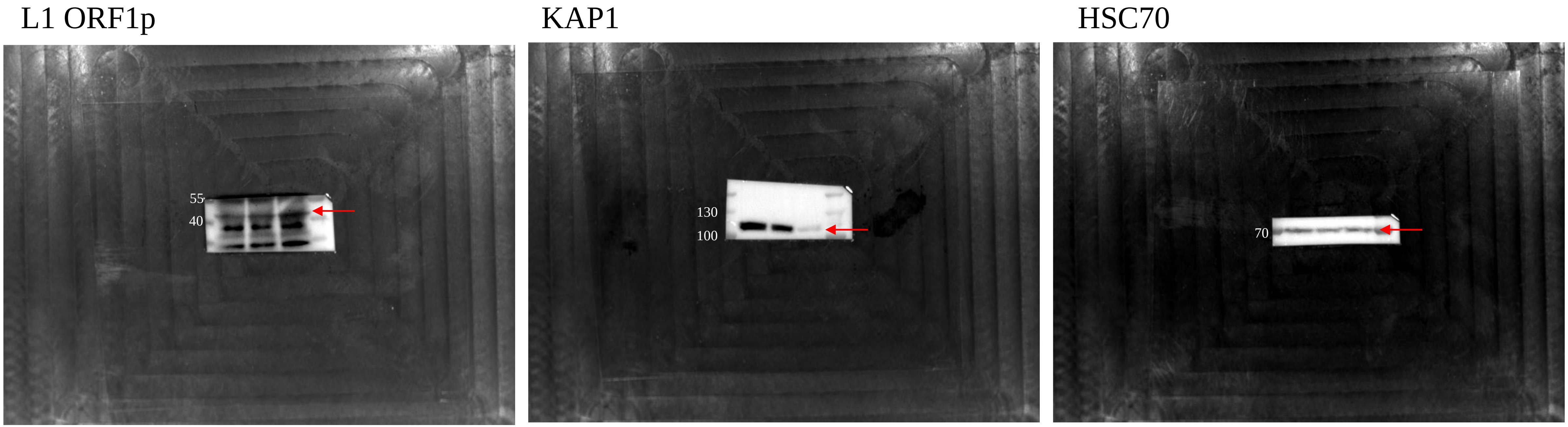

Figure 2B

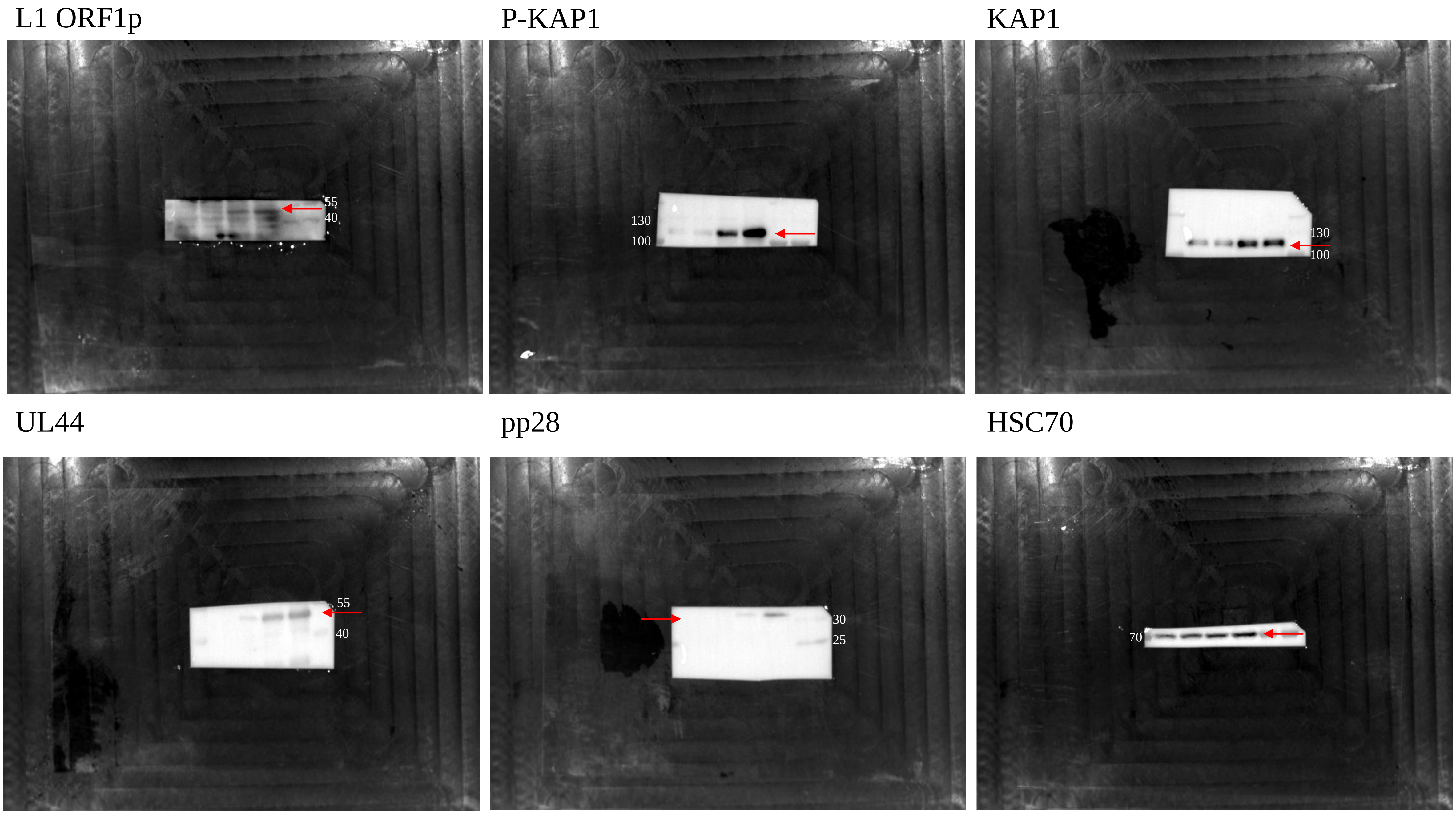

L1 ORF1p
55
40
P-KAP1
130
100
KAP1
130
100
UL44
55
40
pp28
30
25
HSC70
70

Figure 2C

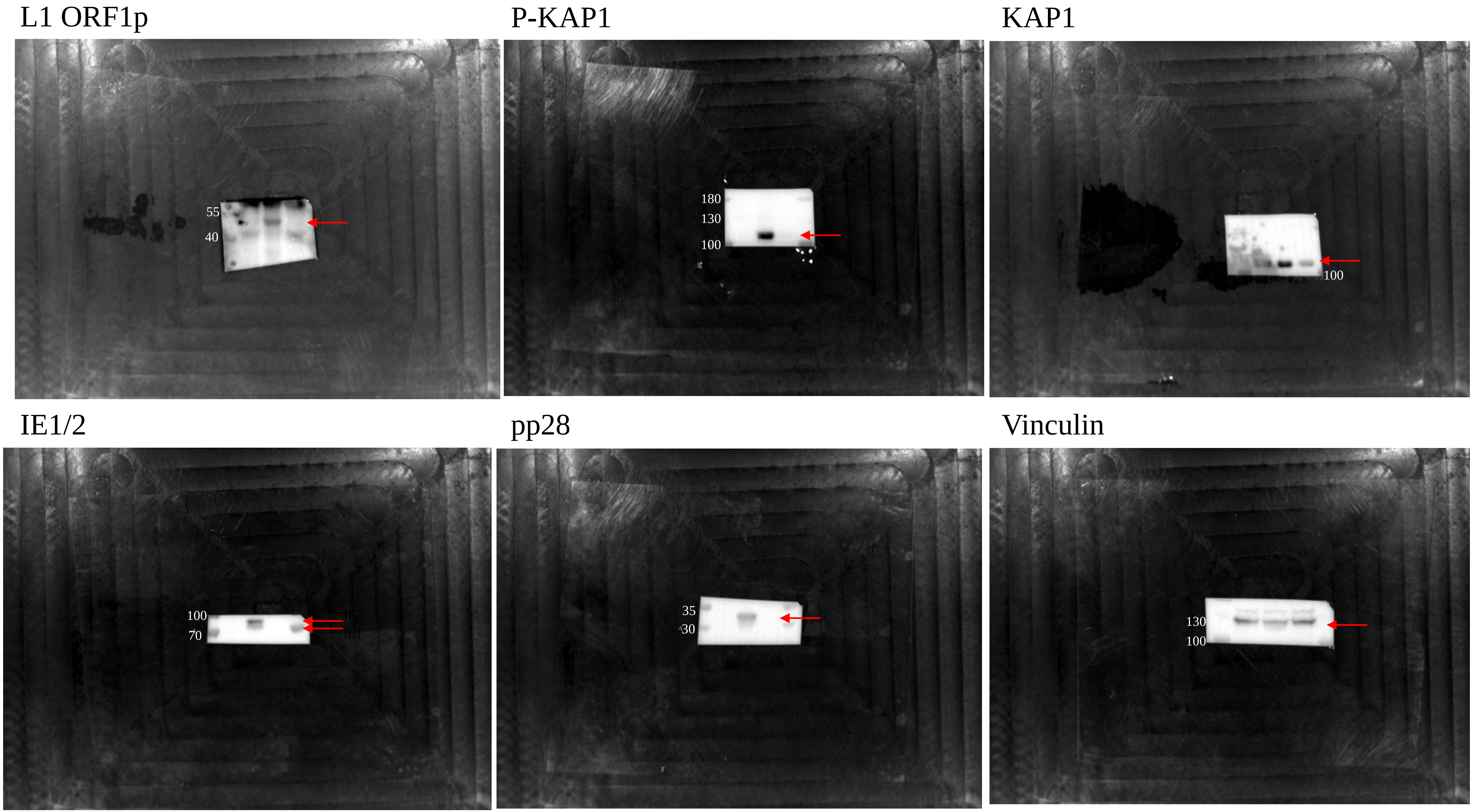

L1 ORF1p
55
40
P-KAP1
180
130
100
KAP1
100
IE1/2
100
70
IE2
IE1
pp28
35
30
Vinculin
130
100

Figure 3A

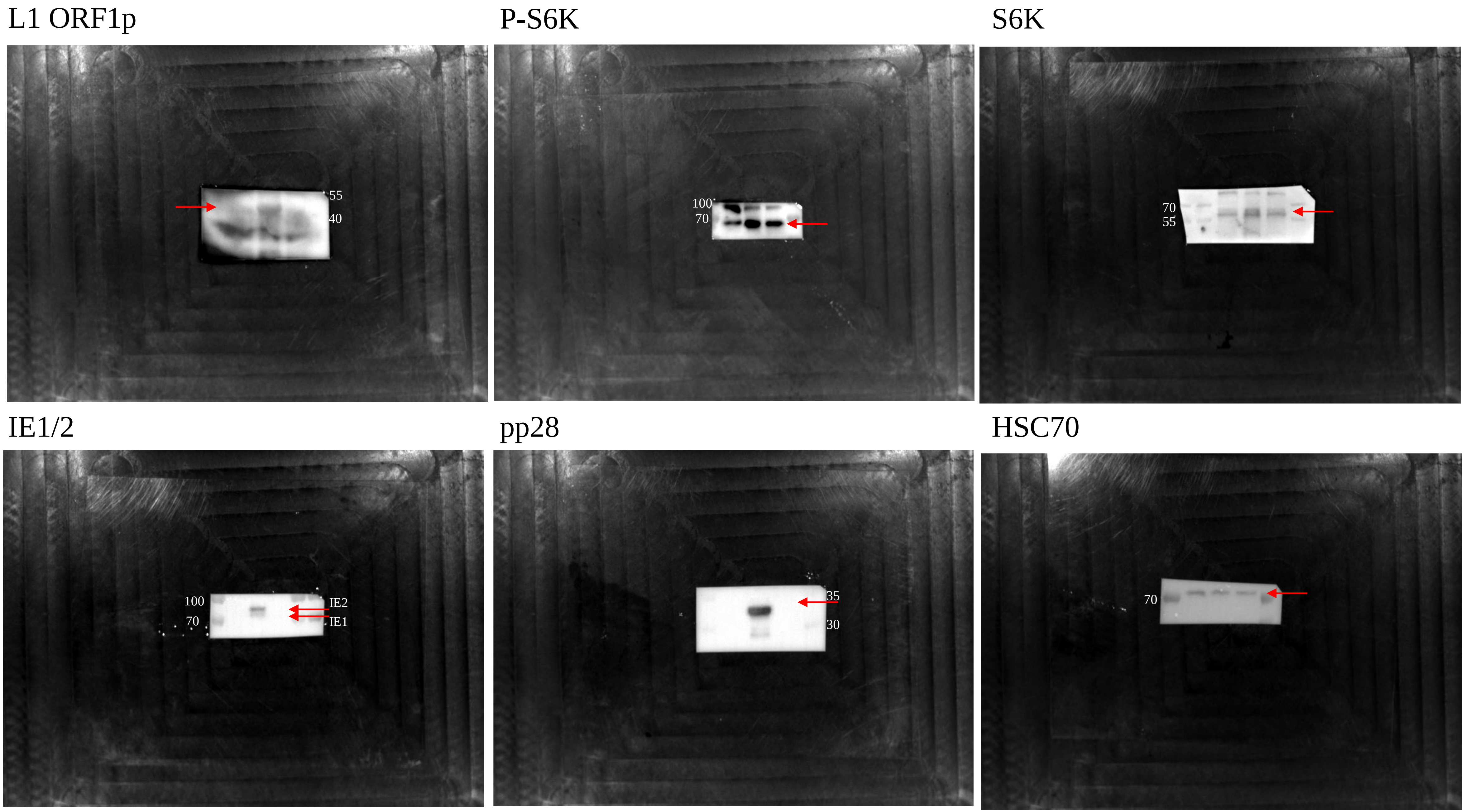

L1 ORF1p
55
40
P-S6K
100
70
S6K
70
55
IE1/2
100
70
IE2
IE1
pp28
35
30
HSC70
70

Figure 3B

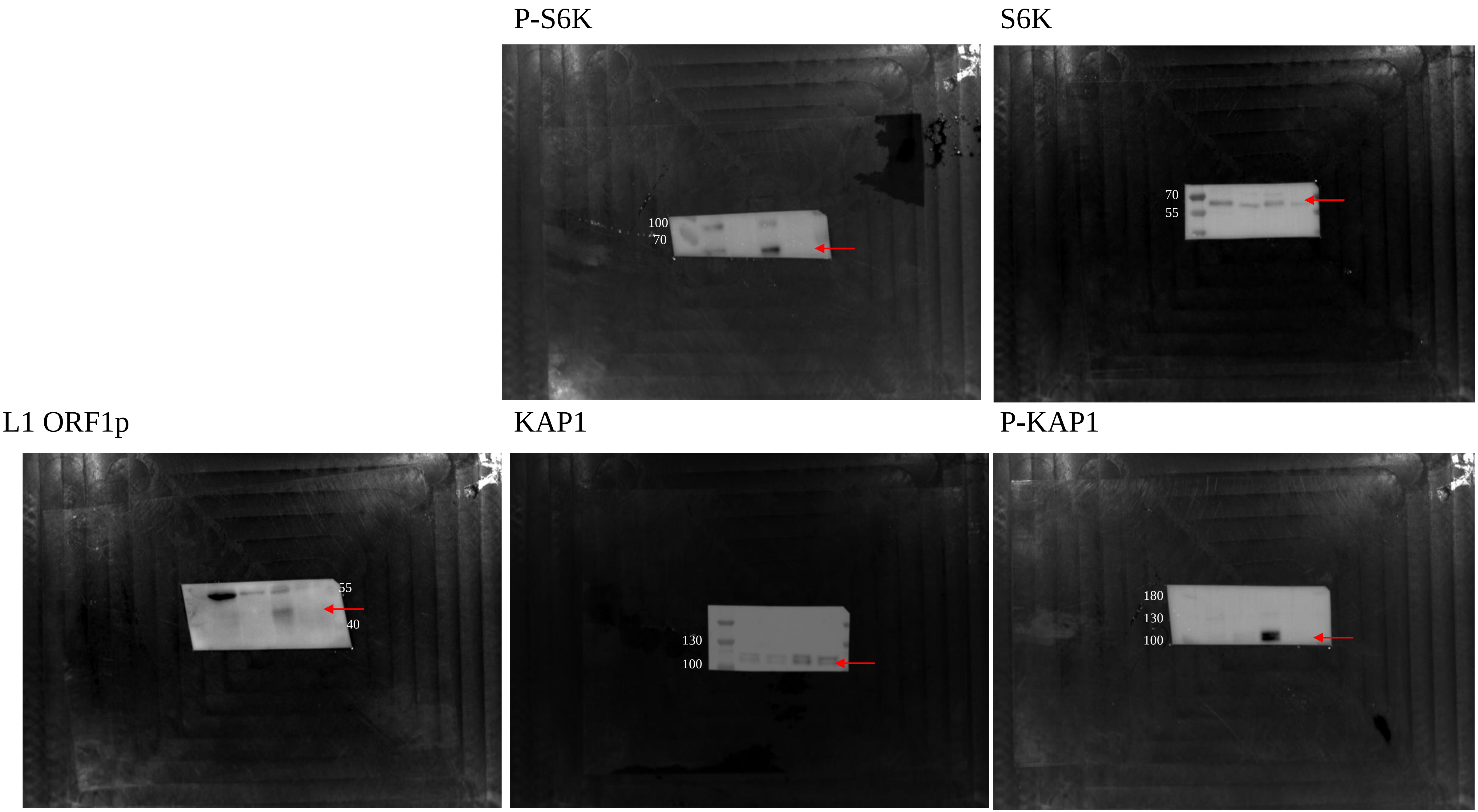
P-S6K
100
70
S6K
70
55
L1 ORF1p
55
40
KAP1
130
100
P-KAP1
180
130
100

Figure 3B

UL44

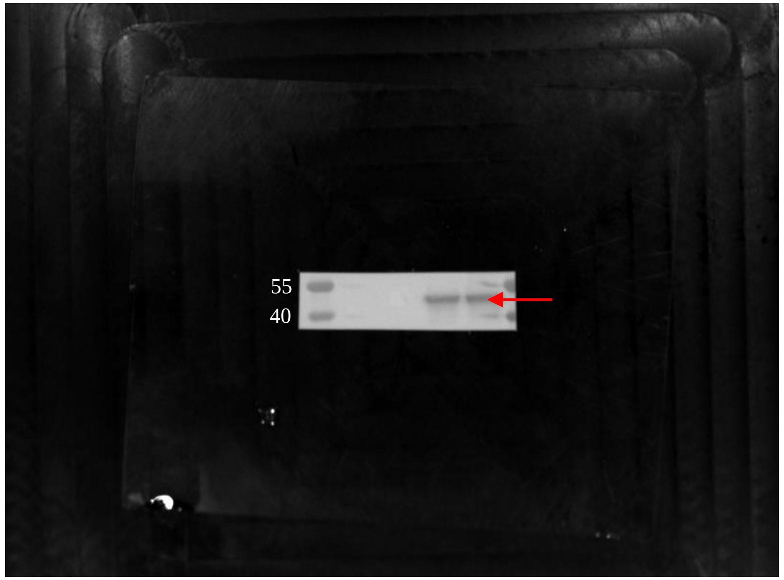

Vinculin

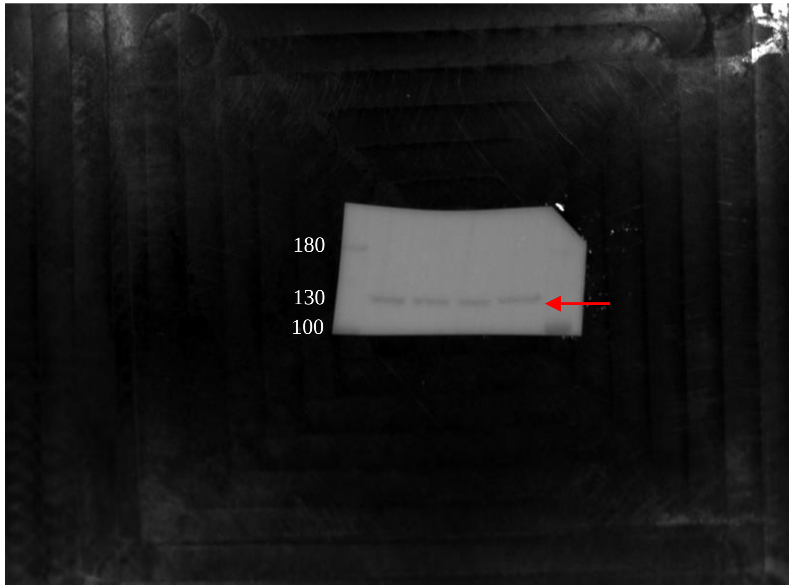

Figure 4D

P-S6K

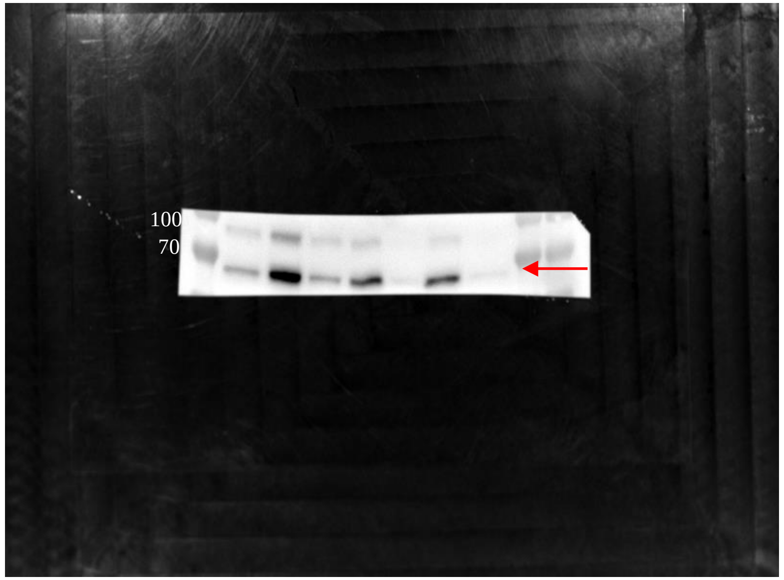

S6K

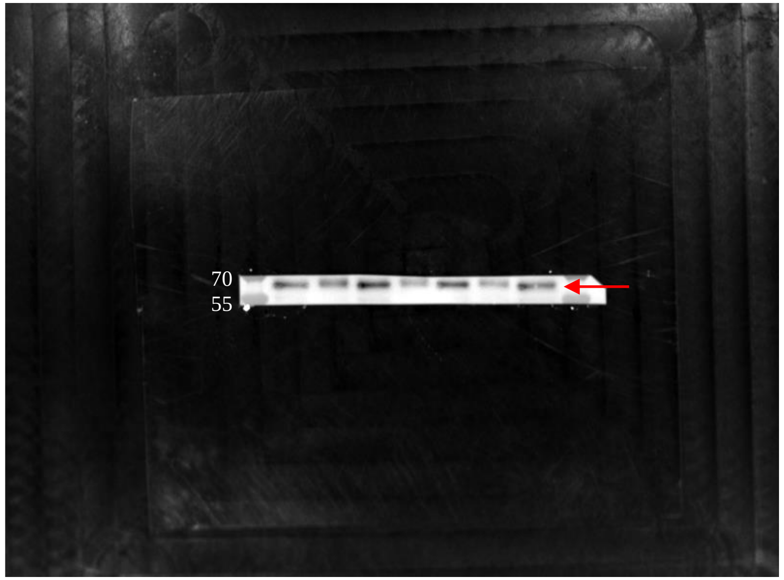

L1 ORF1p

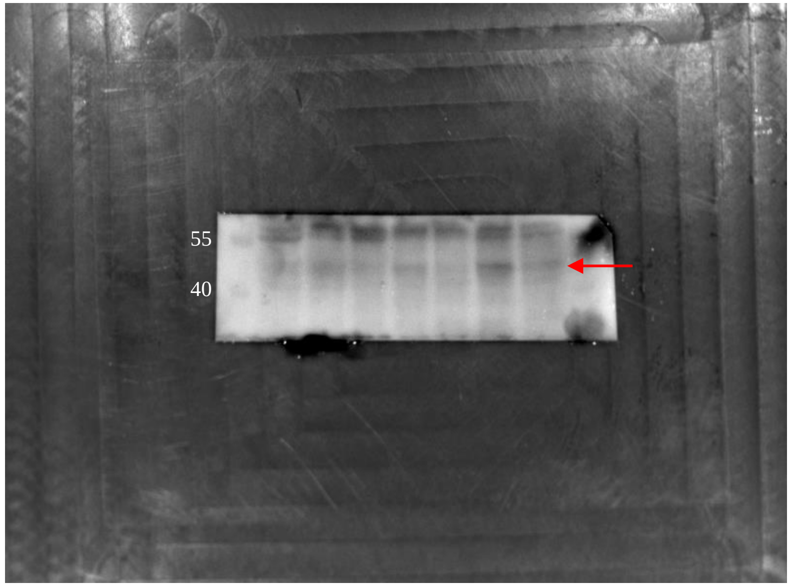

KAP1

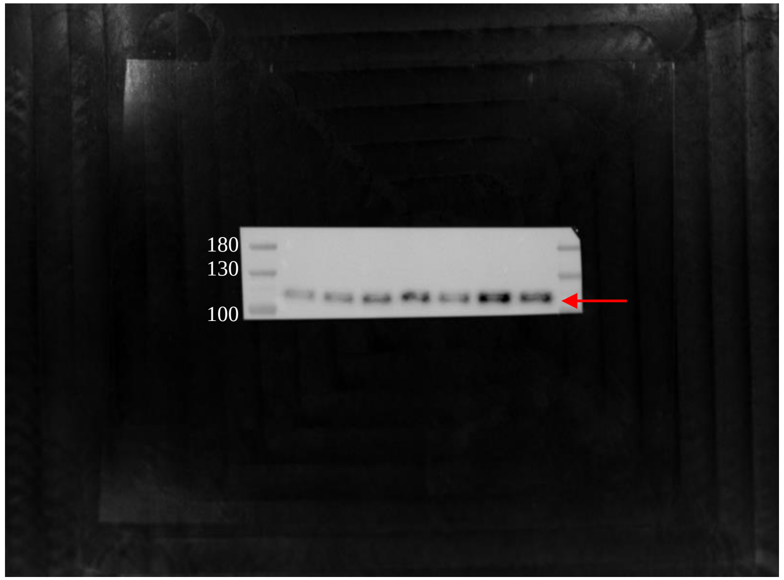

P-KAP1

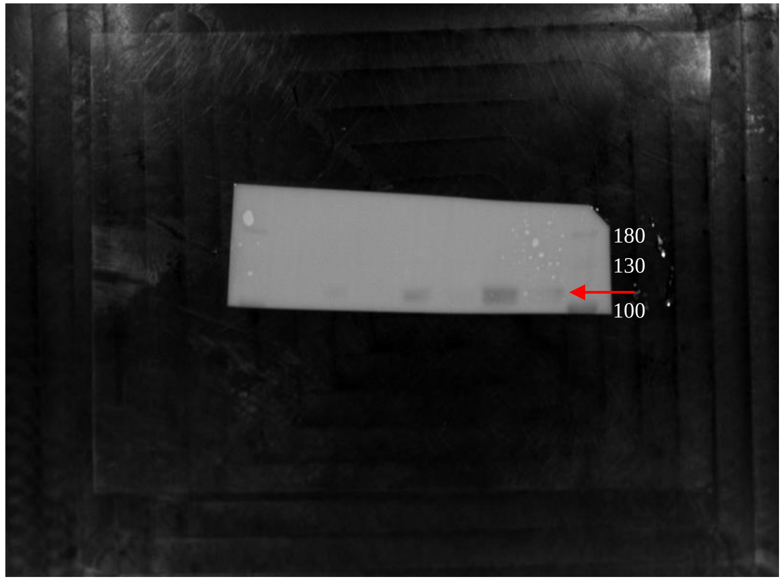

Figure 4D

IE1/2

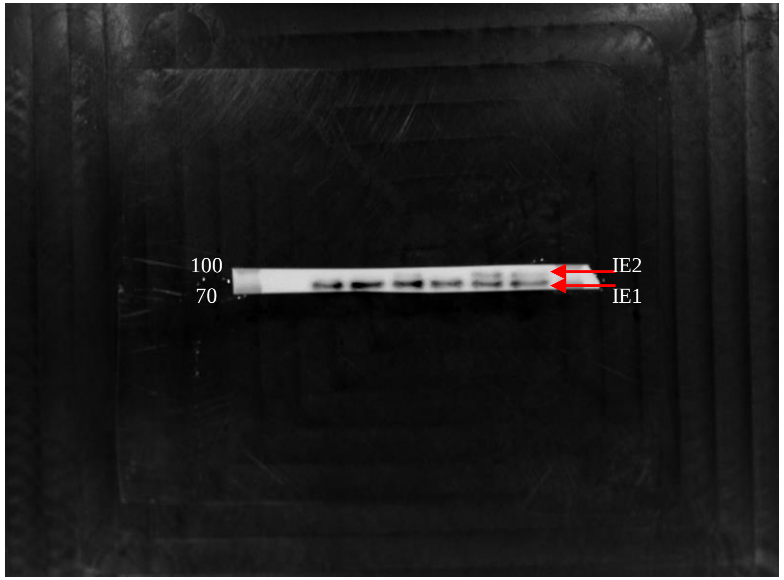

Vinculin

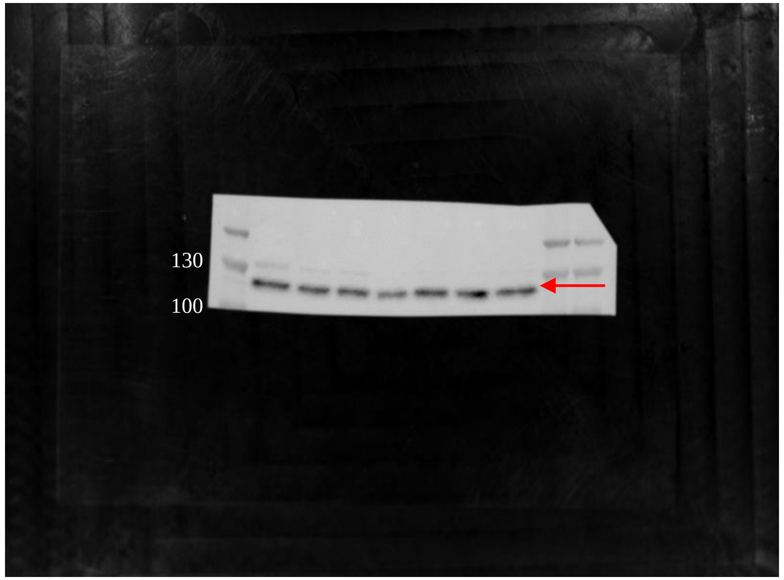

Supplement: S1 File — (PDF) [file pone.0320512.s003.pdf]
