# Supplementary figures and images for: Human cytomegalovirus infection induces L1 expression through UL38-dependent mTOR-KAP1 pathway
Source: PLoS One. 2025 Apr 23;20(4):e0320512. doi: 10.1371/journal.pone.0320512 (PMC12017509; doi:10.1371/journal.pone.0320512)

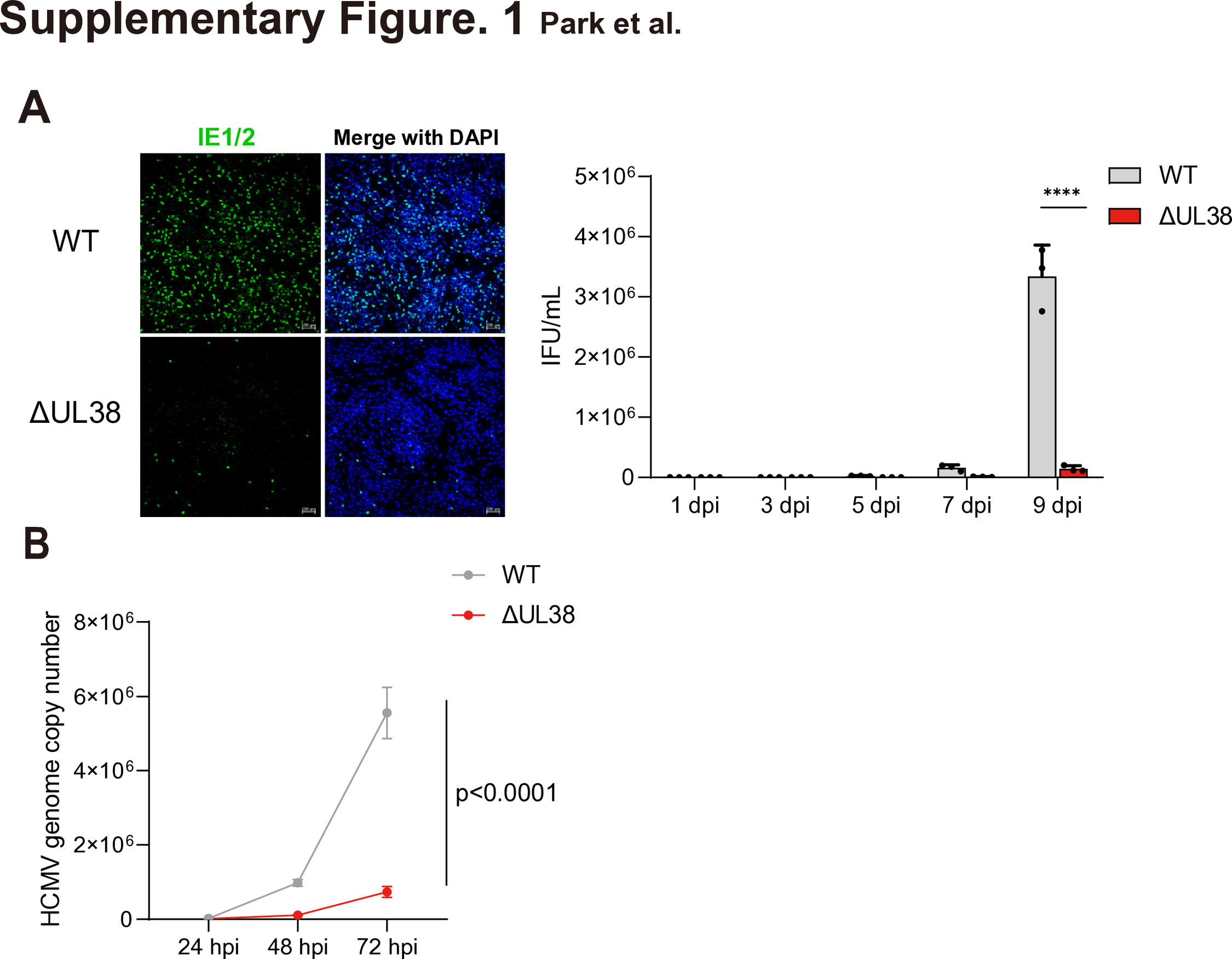

Supplement: S1 Fig — (A) HFF cells were infected with WT and ΔUL38 virus at 0.1 MOI. Cell-free supernatants were harvested at each time point, diluted, and subjected to titration. The image at 9 dpi shows IE1/2 (green) and DAPI (blue). Two-way ANOVA. (B) HFFs were infected with the indicated HCMV at an MOI of 1 for each virus. Viral load was quantified by qPCR using (UL44/MDM2) as targets. Data is presented as mean ± SD from three independent samples (n = 3). Two-way ANOVA. ns p > 0.1234, *p < 0.0332, **p < 0.0023, ***p < 0.0002, ****p < 0.0001. (TIF) [file pone.0320512.s004.tif]
