# Supplementary material for: Human cytomegalovirus infection induces L1 expression through UL38-dependent mTOR-KAP1 pathway
Source: PLoS One. 2025 Apr 23;20(4):e0320512. doi: 10.1371/journal.pone.0320512 (PMC12017509; doi:10.1371/journal.pone.0320512)
Supplement: Attachment — Submitted filename: Response to reviewers.docx [file pone.0320512.s006.docx]

December 3, 2024

Dear Dr. Gianmarco Ferrara,

Thank you for your thoughtful review and for allowing us to revise our manuscript, *“***Human cytomegalovirus infection induces L1 expression through UL38-dependent mTOR-KAP1 pathway***”* (PONE-D-24-53270). We appreciate your detailed feedback and constructive suggestions, which have helped us improve the quality and clarity of our work.

Below, we provide a detailed response to the points raised, categorized by the journal requirements and editorial comments:

**Journal Requirements**

1. **PLOS ONE style requirements:**
   - We have reformatted the manuscript to adhere to the PLOS ONE style templates. The revised manuscript is attached and follows provided formatting guidelines.
   - File names have been updated to comply with the specified naming conventions.
2. **Funding information:**
   - We have ensured that no funding-related text appears in any section of the manuscript. The funding details are provided solely within the Funding Statement section in the online submission form.
3. **Supporting information captions:**
   - Captions for all Supporting Information files have been added to the end of the revised manuscript. Corresponding in-text citations have been updated to maintain consistency throughout the manuscript.
4. **Original blot and gel images:**
   - Full uncropped images underlying all blot results have been included as supplementary files. These images clearly indicate molecular weights and have been formatted following the journal’s requirements.
   - Detailed annotations regarding the blot image data have been provided as per the submission guidelines.

**Editor’s Comments**

We have included all requested full-blot images in supplementary files, ensuring clarity regarding molecular weights and alignment with corresponding controls. These images comply with the journal’s specifications for data presentation and reproducibility.

We trust that these revisions address all the journal requirements and editorial comments comprehensively. If there are additional aspects requiring clarification or further modification, please do not hesitate to contact us.

Thank you again once for your detailed feedback and for considering our manuscript for publication in PLOS ONE.

Sincerely,

Kwangseog Ahn, Professor

Seoul National University

Email: ahnkwangseog@gmail.com
