# Supplementary material for: Human cytomegalovirus infection induces L1 expression through UL38-dependent mTOR-KAP1 pathway
Source: PLoS One. 2025 Apr 23;20(4):e0320512. doi: 10.1371/journal.pone.0320512 (PMC12017509; doi:10.1371/journal.pone.0320512)
Supplement: Attachment — Submitted filename: Response_to_reviewers_auresp_2.docx [file pone.0320512.s007.docx]

February 13, 2025

Dear Dr. Gianmarco Ferrara,

We appreciate the reviewers' valuable feedback and the opportunity to further refine our manuscript titled “**Human cytomegalovirus infection induces L1 expression through the UL38-dependent mTOR-KAP1 pathway**” (PONE-D-24-53270). The constructive suggestions from the reviewers have been instrumental in strengthening the manuscript overall. We have carefully addressed each point raised by the reviewers in our revised manuscript. We present detailed response to each major and minor comment below:

Reviewer #4

**Major concerns:**

1. Western Blots are not quantified, and it is not clear if each Western blot was performed more than once. The authors draw conclusions about changes (or lack of changes) in protein expression, but this should be quantified for the blots shown and a graph should be made from at least three replicate experiments. For example, the authors state that total KAP1 levels “did not decrease” as shown in Fig 2B, but the levels appear to increase. This should be quantified and discussed in the revised manuscript. Quantification of blots needs to be done for Fig 1A-B, 2A-C, 3A-B, 4D.

We thank the reviewer for the valuable comment. Our western blot data repeatedly show increased L1 expression during HCMV infection across different experimental conditions (i.e. HCMV infection, torin treatment, etc). We find the disinhibition of L1 after HCMV infection to be very replicable, which is now quantified with multiple experimental replicates as requested. We performed each western blot analysis at least three times per dataset and quantitatively analyzed the chemiluminescent intensity using ImageJ software. The signal intensity of L1 ORF1p was normalized to the loading controls (either Vinculin or HSC70). The ratio for phosphorylated KAP1 or S6K over the respective total protein level is also indicated as Arabic numeral inside each bar graph (manuscript Figure 2B). These quantifications are now incorporated to Figures 1A-B, 2B-D, 3A-B, and 4D beside a representative image of our western blot data in the revised manuscript.

2. Lines 150-152: “We found that L1 ORF1 protein expression increased throughout the 72-hour HCMV life cycle indicating that L1 expression was upregulated and L1 ORF1 accumulated over the course of HCMV infection (Fig 1A).” At what time points do you observe increased L1 expression? It does not appear to increase at 24hr, but this is not quantified. Is the upregulation at later time points indicative of when during the viral life cycle L1 is induced? Does this correlate with UL38 expression? Is it dependent on viral gene expression, DNA synthesis, etc? These points should be discussed in the manuscript.

Revision Figure 1.

We thank the reviewer for the valuable comment. Western blot analysis shows that L1 protein accumulates over time and the signal is most pronounced after 72 hour post infection (Revision Figure 1A). Although UL38 expression has been reported as early as 6 hpi [1], we think that this apparent delay in L1 protein accumulation is due to multi-step process of HCMV infection-induced L1 disinhibition. Our findings indicate that L1 activation is not directly regulated by UL38 itself but rather occurs through the UL38-mediated activation of the mTOR-KAP1 pathway. After HCMV infection, UL38 upregulates mTOR pathway which then gradually phosphorylates KAP1 and inactivates the repressor. We find that the phosphorylated KAP1 amount is highest at 72hpi which temporally aligns with the upregulation of L1 (Revision Figure 1B). To clarity this point, we revised the manuscript writing and supplemented a clearer quantified bar graphs for our western blot data.

L1 induction is independent of HCMV DNA synthesis. When HFFs were treated with ganciclovir at concentrations of 1, 2, and 4 μM one hour post-infection and harvested at 72 hpi, viral DNA replication was significantly reduced by ganciclovir treatment. However, L1 expression remained largely unchanged compared to the untreated control (Revision Figure 1C).

3. Fig 1B. and 3A. Authors state that UV-HCMV does not induce L1 expression or phospho/total S6K, respectively, but there appears to be a slight induction. This should be quantified and discussed in the revised manuscript.

Revision Figure 2.

We thank the reviewer for the valuable feedback. We have performed a quantitative analysis of the relative chemiluminescent signal from our western blot using ImageJ software and we did not find any noticeable difference in signal intensity between UV-HCMV and non-infected conditions for L1 ORF1p or phosphorylated S6K. There appears to be some basal level signal for phosphorylated S6K but this background is at least two fold lower in intensity than HCMV-infection induced P-S6K signal. Accordingly, we have revised the manuscript to add quantification for each western blot and further clarified this point in the text.

4. Your data is clear that UL38 expression is necessary for driving L1 expression, but is UL38 sufficient to increase mTOR signaling and induction of L1 expression? This could be tested using a plasmid expressing UL38. Or are other HCMV factors required to stimulate L1 expression?

Revision Figure 3.

We thank the reviewer for the valuable comment. To test if UL38 is sufficient to induce L1 disinhibition, we used a luciferase reporter system in HeLa cells to ectopically express HCMV UL38. Our luciferase reporter was designed to express firefly luciferase by L1 promoter and Renilla luciferase driven independently by the SV40 promoter for transfection efficiency normalization. Interestingly upon transient expression of UL38 with the luciferase reporter plasmid, we found a clear increase in firefly luciferase signal after first 24 hours post transfection in HeLa cells, indicating that UL38 expression alone is indeed sufficient to derepress L1 promoter. Furthermore, we confirmed via western blot that the mTOR was activated and KAP1 phosphorylation increased with ectopic UL38 expression in HeLa. However, because HeLa is not permissive for HCMV infection and has extremely low to no expression of endogenous L1, we did not include this data in the manuscript.

5. Previous studies by this group showed that L1 expression during HCMV infection is important for viral replication. This would suggest that a UL38 mutant, which results in reduced L1 levels during infection, would exhibit a growth defect. The authors should provide growth curves for the UL38 mutant virus. Additionally, the authors should provide more discussion about how the findings in this study fit in with the data previously published for L1 expression during HCMV infection.

We thank the reviewer for the valuable comment. As expected, we observed a growth defect of UL38-deleted mutant virus compared to the WT virus when infected at same MOI in HFF cells, as demonstrated by growth curve analysis (Revision Figure 4A). Our previous studies have shown that HCMV utilizes L1 to facilitate viral DNA replication [2]. In line with our previous work, we found that in the absence of UL38, viral DNA replication was impaired (Revision Figure 4B). These data have been added to the supplementary data and we further this finding in the Discussion section.

**Minor concerns:**

1. Fig 1D-E. Is the data RLU or is it relative to non-infected cells (set to 1)? Either is fine, but please describe this in more detail in the figure legend.

We normalized each infection condition to the non-infected control. We modified the data presentation method in Figure 1D and revised the figure legend for improved clarity.

2. The figure legend for Fig 1F refers to up/down panels, but it should be left/right.

We have modified the figure legend of Fig 1F up/down to left/right.

3. Also, for the Fig 1F legend please describe how the data is shown. It is unclear what “relative fold enrichment” means when the results state that the calculated shift in Cq values between nuclease-treated and non-treated samples was calculated.

We have revised the figure legend to provide a more detailed explanation of how the fold enrichment in Fig. 1F was calculated and how the relative fold enrichment was determined through normalization.

4. Lines 56-58 and 334-335 are missing references.

The missing references have been added to the indicated sections in the revised manuscript.

5. One lines 205-208, the %knockdown and %increase in expression are not consistent with the data shown in Fig 2A.

We calculated the average values of KAP1 and L1 ORF1p signal intensity normalized to HSC70 signal intensity and included a relative signal intensity graph displaying the values for each sample in Fig. 2B. Based on these results, we also calculated the percentage difference between siKAP1 and siCon and incorporated it into the Results section.

6. Lines 216-218: “upon HCMV infection, viral factors inactivate...”: you have not thus far shown that it is a viral factor, just infection itself (but not likely entry since you don’t observe changes with UV-HCMV). This could be indirect effects. Please change the statement to something that more accurately reflects the data. Either HCMV infection induces, or HCMV viral gene expression induces… Your data suggests it is at later time points (48-72 hr), suggesting that it is not IE gene expression.

For UV-HCMV, since viral factors are not synthesized *de novo* after host cell entry, we have revised the expression to *‘de novo* translated viral factor'.

7. Fig 2A. legend does not fully explain values quantified. Were signal intensities quantified by ImageJ and normalized to a loading control?

We presented the results by normalizing the signal intensity of L1 ORF1p and KAP1 to the signal intensity of HSC70 in Fig 2B.

8. On lines 267-269, it is stated that HCMV entry was unaffected. This is not exactly what the data show. The data shows equivalent levels of viral DNA, suggesting that there was no defect in entry, viral gene expression, etc. but the authors did not perform an entry assay. Please more accurately describe the results.

In Fig. 4C, we obtained DNA from WT- and ΔUL38-infected HFFs at 3 hpi, a time point when viral DNA replication does not occur, to confirm viral genome entry. Therefore, we have revised the expression from 'viral entry' to 'viral genome entry'.

9. For Fig 4B-C, I cannot determine exactly what was measured based on the figure legend (what UL38 expression is normalized to, how viral DNA was measured). Also, the legend says that 4C shows UL38 expression when it does not. Please make these additions and changes.

We analyzed the expression level of UL38 at the RNA level across each time point in Fig. 4B. In contrast, Fig. 4C focuses on comparing viral genome entry between WT and ΔUL38 HCMV, rather than UL38 expression. To improve clarity, we have refined the figure legend and revised the Results section accordingly.

10. In the discussion, there are multiple mentions of “the HCMV factor”. I’m assuming UL38, but this should be stated more clearly.

We have removed the description of UL38 as a viral factor in the Discussion section and have explicitly referred to it as UL38 for greater clarity.

11. On lines 323-324, it is stated that, “the HCMV factor induces epigenetic derepression by interacting with cellular epigenetic regulator KAP1…” The data does not show a direct interaction, so this statement should be amended to reflect the data.

Since UL38 does not directly phosphorylate KAP1 but instead influences it indirectly through the mTOR pathway, we have replaced 'interacting' with 'modulating' for more accurate wording.

12. On lines 230 and 232, it should read, “the mTOR pathway” and not “mTOR pathway”. Also, on line 239, it should read “KAP1” and not “the KAP1”.

We have inserted 'the' before 'mTOR pathway' and removed 'the' before 'KAP1' for grammatical accuracy.

Sincerely,

Kwangseog Ahn, Professor

Seoul National University

Email: ksahn@snu.ac.kr

**Revision References**

1. Domma AJ, Henderson LA, Goodrum FD, Moorman NJ, Kamil JP. Human cytomegalovirus attenuates AKT activity by destabilizing insulin receptor substrate proteins. J Virol. 2023;97(10):e0056323.

2. Hwang SY, Kim H, Denisko D, Zhao B, Lee D, Jeong J, et al. Human cytomegalovirus harnesses host L1 retrotransposon for efficient replication. Nat Commun. 2024;15(1):7640.
